# A mitochondria-related signature for predicting immune microenvironment and therapeutic response in osteosarcoma

**DOI:** 10.3389/fonc.2022.1085065

**Published:** 2022-12-01

**Authors:** Lina Zhang, Song Wu, Junjie Huang, Yanbin Shi, Yuesong Yin, Xu Cao

**Affiliations:** Department of Orthopaedics, The Third Xiangya Hospital, Central South University, Changsha, China

**Keywords:** osteosarcoma, immune, mitochondria-related signature, therapeutic response, single-cell analysis

## Abstract

**Background:**

Osteosarcoma remains to be the most devastating malignant tumor in children and teenagers. Mitochondria have also been proven to play critical roles in osteosarcoma. However, a mitochondria-related signature has been established in osteosarcoma to comprehensively evaluate the pathogenic roles and regulatory roles of mitochondria in osteosarcoma.

**Methods:**

In this study, osteosarcoma samples' transcriptome data and clinical information were collected from Therapeutically Applicable Research to Generate Effective Treatments (TARGET) and Gene Expression Omnibus (GEO) databases. A comprehensive bioinformatics analysis was performed on the samples at the bulk RNA sequencing level and single-cell RNA sequencing (scRNA-seq) level. EdU, Transwell, and immunohistochemistry (IHC) were performed on PCCB.

**Results:**

A mitochondria-related signature was constructed in osteosarcoma patients. The prognostic value of the mitochondria-related signature was explored. The predictive value of the mitochondria-related signature in the immune microenvironment and chemotherapy agents was explored. The association between mitochondria and immunity in the tumor microenvironment of osteosarcoma at the scRNA-seq level was investigated. The tumorigenic role of the critical mitochondria-related gene, PCCB, was verified by *in vitro* validation.

**Conclusion:**

In conclusion, a mitochondria-related signature was developed in osteosarcoma with solid predictive values in the immune microenvironment, chemotherapy agents, and prognosis.

## Introduction

Osteosarcoma remains the most devastating malignant tumor in children and teenagers (1). Osteosarcoma develops from the mesenchymal cell line, and the rapid growth of the cancer is due to the direct or indirect formation of tumor osteoid tissue and bone tissue during the chondral stage (1). The closer the tumor site is to the trunk, the higher the mortality. The key factors affecting the prognosis are early diagnosis, complete tumor resection, chemotherapy, and radiotherapy before and after surgery. In addition, it is also related to the tissue type and size of tumor cells, the increase of serum alkaline phosphatase before and after surgery, and whether local lymph nodes are involved (2). The primary treatment for osteosarcoma is radical surgical resection. Consolidation of chemical or radiation therapy after tumor resection is significant for controlling tumor metastasis and improving survival rate (3). Immunotherapy involves the intravenous infusion of lymphocytes or interferon and transfer factors, but the efficacy is uncertain (4).

Mitochondria have been well recognized as a critical mediator for oncogenesis. Based on their function as major bioenergy promoters, mitochondria are actively involved in regulating tumor anabolism, controlling REDOX and calcium homeostasis, participating in transcriptional regulation, and controlling cell death. Mitochondrial dysfunction leads to the release of cytochrome C, the production of mitochondrial reactive oxygen species (mtROS), and the generation of metabolites, further initiating signaling cascades that affect gene expression, cell activation, cell proliferation, and cell differentiation (5, 6). Mitochondria may promote malignant transformation through three main mechanisms: (1) Reactive oxygen species (ROS), mainly derived from the mitochondrial respiratory chain, contribute to the accumulation of potential oncogenic DNA defects, and the activation of potential oncogenic signaling pathways (7); (2) Abnormal accumulation of some mitochondrial metabolites, including fumaric acid, succinic acid, and 2-hydroxyglutaric acid (2-Hg) (8); (3) Defective mitochondrial permeability transition (MPT) function promotes the formation of malignant precursors (9).

Mitochondria have also been proven to play critical roles in osteosarcoma. AICAR was reported to induce mitochondrial apoptosis in osteosarcoma through an AMPK-dependent pathway (10). The mitochondrial BIG3-PHB2 complex formation was reported to promote the survival and proliferation of osteosarcoma (11). Besides, targeting autophagy was reported to enhance atezolizumab-induced mitochondria-related apoptosis in osteosarcoma (12). Mitochondria-regulated cell death and energetic metabolism are intimately connected in osteosarcoma (13). However, a mitochondria-related signature has never been established in osteosarcoma to comprehensively evaluate the pathogenic roles and regulatory roles of mitochondria in osteosarcoma. More importantly, the interconnection between mitochondria and the tumor microenvironment of osteosarcoma remains to be deciphered.

In this study, osteosarcoma samples’ transcriptome data and clinical information were collected from Therapeutically Applicable Research to Generate Effective Treatments (TARGET) and Gene Expression Omnibus (GEO) databases. A mitochondria-related signature was constructed in osteosarcoma patients. The prognostic value of the mitochondria-related signature was explored. The predictive value of the mitochondria-related signature in the immune microenvironment was explored. The predictive value of the mitochondria-related signature in chemotherapy agents was explored. The association between mitochondria and immunity in the tumor microenvironment of osteosarcoma at the single-cell RNA sequencing (scRNA-seq) level was investigated. The tumorigenic role of the critical mitochondria-related gene, PCCB, was verified by *in vitro* validation. To the best of our knowledge, this is the first study assessing the effect of mitochondria on the prognosis, immune microenvironment, and therapeutic efficacy in osteosarcoma.

## Materials and methods

This study was ethically approved by the institutional review board (IRB) of the Third Xiangya Hospital, Central South University (No: 2020-S221). All experiments involving human tissues were performed based on guidelines approved by the IRB. A signed informed consent form was obtained from each patient.

### Data collection and procession

84 osteosarcoma samples with transcriptome data and clinical information were accessed from the TARGET database ((https://xenabrowser.net/) and were designed as the training cohort. 53 osteosarcoma samples with transcriptome data and clinical information were accessed from the GEO database (https://www.ncbi.nlm.nih.gov/geo/) and were designed as the validating cohort (GSE21257). Samples with less than one month follow-up time and a lack of overall survival information were excluded. The count data were normalized using the R package ‘DEseq2’. The scRNA-seq data of osteosarcoma samples (primary osteosarcoma lesions, ‘BC2’ and ‘BC3’) were accessed from GSE152048 in the GEO database. The mitochondria-related genes were accessed from the MitoCarta3.0 database (http://www.broadinstitute.org/mitocarta).

### Construction of the mitochondria-related signature

The univariate Cox regression analysis was performed on the mitochondria-related genes to determine their prognostic values. The least absolute shrinkage and selection operator (LASSO) regression analysis was performed on the prognostic mitochondria-related genes for dimension reduction. The stepwise multivariate Cox regression analysis was further performed on the prognostic mitochondria-related genes for dimension reduction. A mitochondria-related signature was developed based on the following formula: Risk Score=∑*Expression*(*Gene*)×*Coefficient* .

### Prognostic value of the mitochondria-related signature

The survival curves were generated using the R package ‘survival’. The survival curves regarding different clinical factors were developed independently. The receiver operating characteristic (ROC) curve was generated using the R package ‘timeROC’, and the area under the curve (AUC) value was calculated.

### Predictive value of the mitochondria-related signature in the immune microenvironment

The single-sample gene-set enrichment analysis (ssGSEA) algorithm was used to quantify the abundance of 28 specific immune cell types using the R package ‘GSVA’ (14). The Estimated Stromal and Immune cells in Malignant Tumor tissues using Expression data (ESTIMATE) algorithm was used to determine the microenvironment scores using the R package ‘estimate’ (15).

### Functional annotation of the mitochondria-related signature

The differentially expressed genes (DEGs) between two mitochondria-related signature score groups were determined. The DEGs were visualized by volcano plot using the R package ‘EnhancedVolcano’. The DEGs were visualized by heatmap using the R package ‘pheatmap’. The gene sets of ‘c2.cp.kegg.v7.4.symbols’ and ‘c5.go.bp.v7.4.symbols’ were obtained from MSigDB database for performing the gene set variation analysis (GSVA). Gene Ontology (GO) and Kyoto Encyclopedia of Genes and Genomes (KEGG) enrichment analysis was conducted with the R package ‘clusterProfiler’ and ‘org.Hs.eg.db’.

### Prediction value of the mitochondria-related signature in chemotherapy agents

The transcriptome data and drug response information of over 1,000 cancer cell lines were collected from the Genomics of Drug Sensitivity in Cancer (GDSC, http://www.cancerrxgene.org/) database. The mitochondria-related signature was developed in each cancer cell line. The Spearman method was used to evaluate the correlation between risk score and half-maximal inhibitory concentration (IC50) of each cancer cell line.

### scRNA-seq analysis of the mitochondria-related signature

The scRNA-seq matrix of primary osteosarcoma samples from GSE152048 was processed using the R package ‘Seurat’. The function ‘NormalizedData’ was used to normalize the scRNA-seq data. The function ‘FindVariableFeatures’ was used to identify the 1,000 most variable genes. The function ‘RunPCA’ was used for dimension reduction. A K-nearest neighbor was analyzed using the function ‘FindNeighbors’, and the cells were combined with the function ‘FindClusters’. The function Uniform Manifold Approximation and Projection for Dimension Reduction (UMAP) was used for visualization. All cells were annotated using the R package ‘Single R’. The function ‘FindMarkers’ was used to find DEGs between two mitochondria-related signature score groups of osteosarcoma cells. The pseudotime trajectory analysis was performed using the R package ‘monocle’ (16). GO and KEGG enrichment analysis was conducted with the R package ‘clusterProfiler’ and ‘org.Hs.eg.db’. The cell communication pattern was explored using the R package ‘iTalk’.

### Immunohistochemistry

Three pairs of formalin-fixed paraffin-embedded osteosarcoma tissue and para-carcinoma tissue blocks from three osteosarcoma patients (post-chemotherapy) were collected and used for 5 μm paraffin sections. IHC was performed following the manufacturer’s protocol of the Rabbit-enhanced polymer method detection system (ZSGB-BIO, PV-9000, China). The slides were deparaffinized and rehydrated using xylene and gradient-concentration ethyl alcohol. The antigen retrieval was performed with sodium citrate at 95°C. The slides were blocked using an endogenous peroxidase blocker for 10 min at room temperature. Samples were incubated with primary antibody against PCCB (127549, Zenbio, China) overnight at 4°C, reaction enhancer for 20 min at 37°C, and enhanced enzyme-conjugated sheep anti-rabbit IgG polymer for 20 min at 37°C. The slides were stained with 3, 30-diaminobenzidine tetrahydrochloride (DAB) and counterstained with hematoxylin.

### Cell culture

Two osteosarcoma cell lines (U2OS and MNNG/HOS) were obtained from the Procell Life Science & Technology Co., Ltd. U2OS and MNNG/HOS were correspondingly cultured in McCoy’s 5A (Procell, China) and MEM (Procell, China) supplemented with 10% fetal bovine serum (FBS, Gibco, USA) and 1% penicillin-streptomycin solution (Biosharp, China) at 37°C with saturated humidity and 5% CO2. The average time of culture medium exchange was 24-48h. The cells were digested with trypsin-EDTA (Gibco, USA) and passaged when cell adhesion exceeded 80% confluency.

### Small interfering RNA transfection

The PCCB siRNA (si-PCCB) and the nonspecific control siRNA (si-NC) were synthesized by JTSBio (Wuhan, China). The siRNAs sequences are as follows: PCCB-1 (F: CCCUAACAGACUUCACGUUTT R: AACGUGAAGUCUGUUAGGGTT), PCCB-2 (F: CCAAGCUUCUCUACGCAUUTT R: AAUGCGUAGAGAAGCUUGGTT), PCCB-3 (F: CCGCAGAGAUUGCAGUCAUTT R: AUGACUGCAAUCUCUGCGGTT). The siRNAs were transfected into U2OS and MNNG/HOS cells using a jetPRIME transfection reagent (Polyplus, France). RNA extraction was performed 48h after transfection.

### Real-time quantitative polymerase chain reaction

The primer sequences are as follows: PCCB (F: TGTCTTCAGTCAGGATTTTACAGTT R: GGCCTGGTCCATGATTTTGC), GAPDH (F: AATGGGCAGCCGTTAGGAAA R: GCCCAATACGACCAAATCAGAG). Total RNA from cultured cells was extracted using Rnafast200 (Fastagen, Japan), and cDNA was synthesized using HiScript II Q RT SuperMix for qPCR (Vazyme, China). ChamQ Universal SYBR qPCR Master Mix (Vazyme, China) was used to conduct RT-qPCR based on the manufacturer’s protocol. All steps for the RT-qPCR reaction were conducted as follows: initial denaturation at 95°C for 30s, one cycle; denaturation at 95°C for 10s, 40 cycles; dissolution curve at 95°C for 15s, 60°C for 60s, 95°C for 15s, one cycle. Gene expression levels were normalized to those of GAPDH and calculated using lg2–△△Ct method.

### Western blot

A mixture of RIPA (Beyotime, China) and PMSF (Beyotime, China) was used to lyse U2OS and MNNG/HOS cells for protein extraction. Loading Buffer (Biosharp, China) was added to the protein supernatant, and then the sample was boiled to denature the protein. Then proteins were separated using SDS–PAGE gel (Biosharp, China), transferred to PVDF membranes (Millipore, USA), and blocked in 5% skimmed milk for 1 h. Then membranes were incubated overnight at 4°C with primary antibodies, including PCCB (127549, Zenbio, China) and GAPDH (10494-1-AP, Proteintech, China). The membranes were incubated with HRP-conjugated secondary antibody (SA00001-2, Proteintech, China) the following day. Protein bands were captured with a UVP Chem studio PLUS 815 (Analytik Jena, Germany).

### EdU assay

Proliferating U2OS and MNNG/HOS cells were identified using the EdU Imaging Kits (APExBIO, USA), and cell nuclei were stained using Hoechst (Invitrogen, USA). Image Pro-Plus version 6.0 (Media Cybernetics, USA) was used for counting EdU-positive cells.

### Statistical analysis

All bioinformatics statistical analyses were performed using the R project (version 4.0.3, https://www.r-project.org/). The Wilcoxon and One-way ANOVA tests were used to compare the difference among groups. All statistical analyses in the cell experiment are based on mean ± SD using Graphpad Prism (version 8.0.2.263). The Benjamini-Hochberg method was used to obtain adjusted p values. The adjusted p value< 0.05 was considered statistically significant.

## Results

### Construction of the mitochondria-related signature

**Figure 1:**
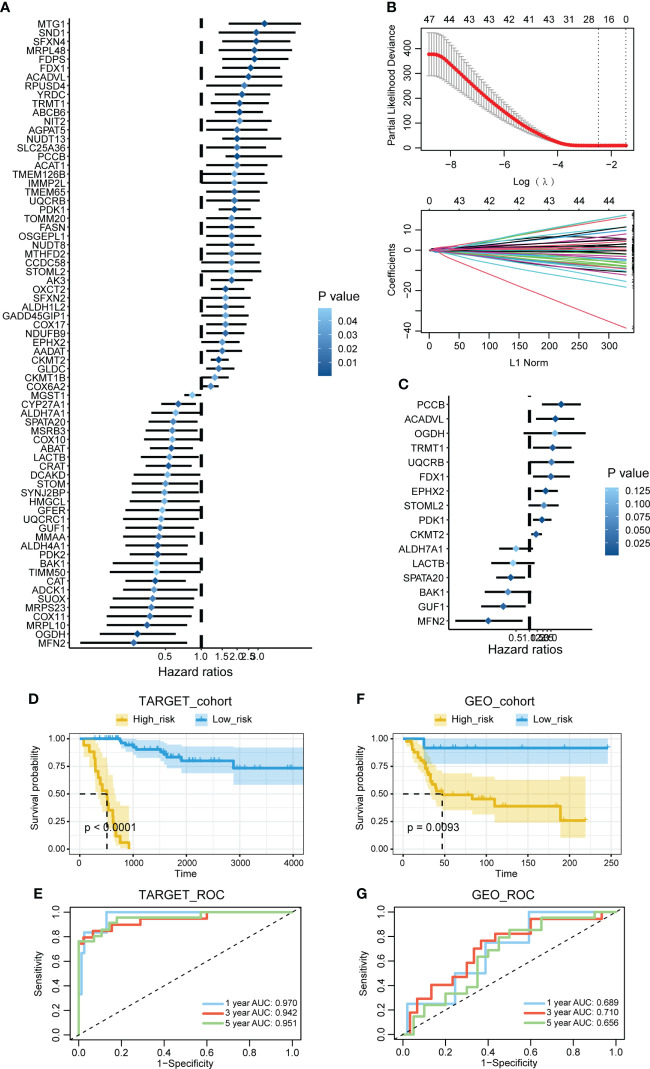
Construction of the mitochondria-related signature. **(A)** The univariate Cox regression analysis was performed on the mitochondria-related genes. **(B)** The LASSO regression analysis was performed on the prognostic mitochondria-related genes. **(C)** The stepwise multivariate Cox regression analysis was performed on the prognostic mitochondria-related genes. **(D)** Survival analysis was performed on the two mitochondria-related signature score groups in TARGET. **(E)** Survival analysis was performed on the two mitochondria-related signature score groups in GSE21257. **(F)** The 1-year, 3-year, and 5-year ROC curves of the mitochondria-related signature in TARGET. **(G)** The 1-year, 3-year, and 5-year ROC curves of the mitochondria-related signature in GSE21257.

The univariate Cox regression analysis was performed on the mitochondria-related genes to determine their prognostic values (**Figure 1A**). The LASSO regression analysis was performed on the prognostic mitochondria-related genes for dimension reduction (**Figure 1B**). The stepwise multivariate Cox regression analysis was further performed on the prognostic mitochondria-related genes for dimension reduction (**Figure 1C**). Survival analysis was performed on the prognostic mitochondria-related genes, among which nine genes predicted worse survival and eight genes predicted better survival (**Figure S1**). The mitochondria-related signature was developed based on the sum of the expression values of the prognostic mitochondria-related genes and their corresponding coefficients. The formula is as follows: OGDH x (1.299)+GUF1 x (-1.34)+FDX1 x (1.115)+ACADVL x (1.335)+PCCB x (1.635)+PDK1 x (0.658)+STOML2 x (0.727)+LACTB x (-0.852)+UQCRB x (1.145)+MFN2 x (-2.086)+CKMT2 x (0.368)+ALDH7A1 x (-0.688)+TRMT1 x (1.189)+EPHX2 x (0.841)+BAK1 x (-1.113)+SPATA20 x (-0.958).

### Prognostic value of the mitochondria-related signature

**Figure 2:**
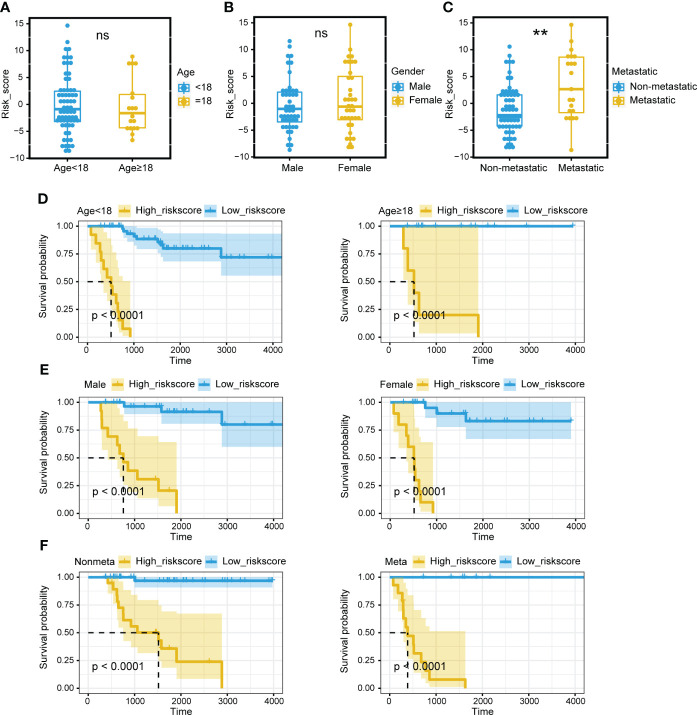
Prognostic value of the mitochondria-related signature. **(A)** The different levels of the mitochondria-related signature score regarding age. **(B)** The different levels of the mitochondria-related signature score regarding gender. **(C)** The different levels of the mitochondria-related signature score regarding metastasis. **(D)** Survival analysis was performed on the two mitochondria-related signature score groups regarding age in TARGET. **(E)** Survival analysis was performed on the two mitochondria-related signature score groups regarding gender in TARGET. **(F)** Survival analysis was performed on the two mitochondria-related signature score groups regarding metastasis in TARGET. ns, no significance; **p < 0.01.

Survival analysis was performed on the two mitochondria-related signature score groups in TARGET, and the high score group was associated with shortened survival time (**Figure 1D**). Survival analysis was also performed on the two mitochondria-related signature score groups in GSE21257, and the high score group was associated with shortened survival time (**Figure 1E**). The 1-year, 3-year, and 5-year ROC curves had values of 0.97, 0.942, and 0.951 in TARGET, while the 1-year, 3-year, and 5-year ROC curves had values of 0.689, 0.71, and 0.656 in GSE21257 (**Figures 1F, G**). The mitochondria-related signature score was not significantly different between the two age groups (**Figure 2A**). The mitochondria-related signature score was not significantly different between the two gender groups (**Figure 2B**). Notably, the mitochondria-related signature score was significantly different between the metastatic group and the non-metastatic group (**Figure 2C**). In osteosarcoma patients with age< 18, the high score group was associated with shortened survival time (**Figure 2D**). Likewise, in osteosarcoma patients with age > 18, the high score group was associated with shortened survival time (**Figure 2D**). In male osteosarcoma patients, the high score group was associated with shortened survival time (**Figure 2E**). Likewise, in osteosarcoma patients, the high score group was associated with shortened survival time (**Figure 2E**). In metastatic osteosarcoma patients, the high score group was associated with shortened survival time (**Figure 2F**). Likewise, in non-metastatic osteosarcoma patients, the high score group was associated with shortened survival time (**Figure 2F**).

### Predictive value of the mitochondria-related signature in the immune microenvironment

**Figure 3:**
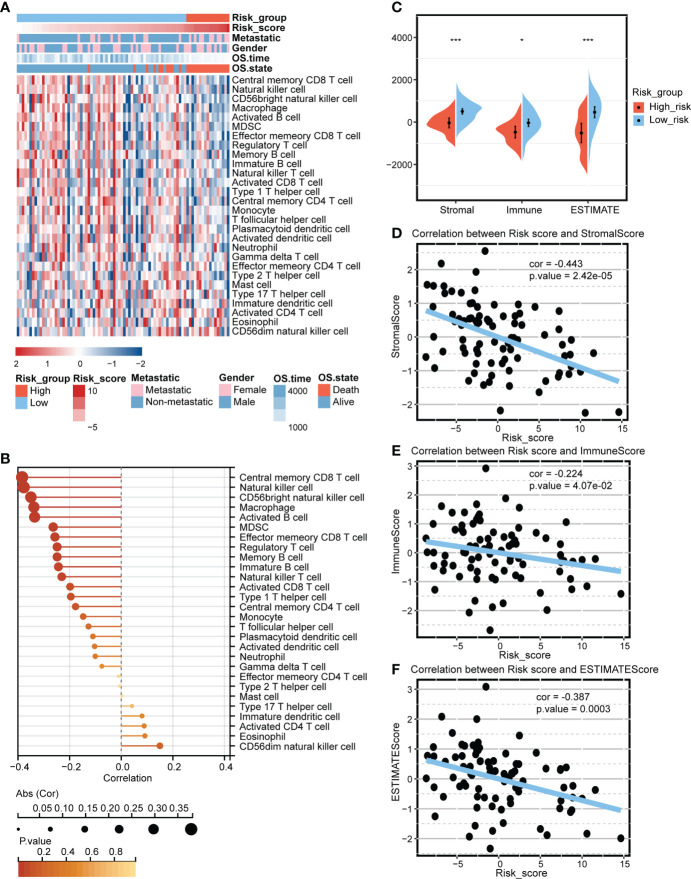
Predictive value of the mitochondria-related signature in the immune microenvironment. **(A)** Heatmap depicting the association between the mitochondria-related signature and immune cells. **(B)** Dot plot depicting the association between the mitochondria-related signature and immune cells. **(C)** The different levels of the mitochondria-related signature score regarding microenvironment scores. **(D)** The association between the mitochondria-related signature and stromal score. **(E)** The association between the mitochondria-related signature and immune score. **(F)** The association between the mitochondria-related signature and ESTIMATE score. *p < 0.05; ***p < 0.001.

The mitochondria-related signature was negatively associated with multiple immune cells, including T cell, B cell, natural killer T cell, macrophage, mast cell, and neutrophil (**Figure 3A**). Notably, central memory CD8 T cell, natural killer cell, CD56bright natural killer cell, macrophage, and activated B cell were the top five cells highly correlated with the mitochondria-related signature (**Figure 3B**). The high score group was associated with lower levels of microenvironment scores, including stromal score, immune score, and ESTIMATE score (**Figure 3C**). Besides, the mitochondria-related signature was negatively associated with stromal score (**Figure 3D**), immune score (**Figure 3E**), and ESTIMATE score (**Figure 3F**).

### Functional annotation of the mitochondria-related signature

**Figure 4:**
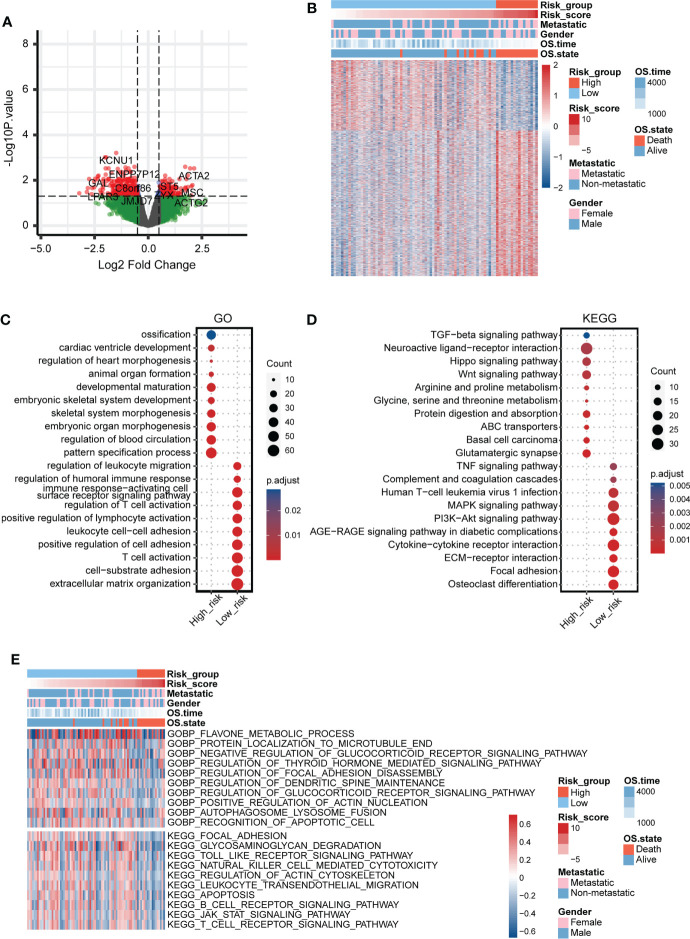
Functional annotation of the mitochondria-related signature. **(A)** Volcano plot for the DEGs between the two mitochondria-related signature score groups. **(B)** Heatmap for the DEGs between the two mitochondria-related signature score groups. **(C)** GO enrichment analysis for the DEGs between the two mitochondria-related signature score groups. **(D)** KEGG enrichment analysis for the DEGs between the two mitochondria-related signature score groups. **(E)** GSVA for the DEGs between the two mitochondria-related signature score groups.

The DEGs between the two mitochondria-related signature score groups were identified (**Figure 4A**). The distribution of the DEGs between the two mitochondria-related signature score groups is shown in **Figure 4B**. GO enrichment analysis was performed on the DEGs (**Figure 4C**). Ossification, embryonic skeletal system development, and pattern specification process were highly enriched in the high score group. T cell activation, extracellular matrix organization, and leukocyte cell-cell adhesion were highly enriched in the low score group. KEGG enrichment analysis was performed on the DEGs (**Figure 4D**). TGF-β signaling pathway, hippo signaling pathway, and wnt signaling pathway were highly enriched in the high score group. Cytokine-cytokine receptor interaction, ECM-receptor interaction, and focal adhesion were highly enriched in the low score group. Besides, GSVA of GO and KEGG pathways confirmed that autophagosome-lysosome fusion, recognition of apoptotic cell, T cell receptor signaling pathway, and B cell receptor signaling pathway were negatively associated with the mitochondria-related signature (**Figure 4E**).

### Prediction value of the mitochondria-related signature in chemotherapy agents

The potential value of the mitochondria-related signature in predicting chemotherapy agents was explored based on the GSDC database. The correlation between IC50 of drugs and the mitochondria-related signature in cancer cell lines was explored. The drug sensitivity of 30 drugs was identified to be significantly associated with the mitochondria-related signature (**Figure S2A**). Besides, the targeted signaling pathways of these drugs were exhibited (**Figure S2B**). 24 drugs were negatively associated with the mitochondria-related signature, including apoptosis regulation inhibitor AZD5991, protein stability and degradation inhibitor ML323, and kinases inhibitor BMS-345541. In addition, six drugs were positively associated with the mitochondria-related signature, including ERK MAPK signaling inhibitor Refametinib, RTK signaling inhibitor NVP-TAE684, and kinases inhibitor A-770041. The overall predicted drug sensitivity and drug resistance in targeted signaling pathways are shown in **Figure S2C**.

### scRNA-seq analysis for the mitochondria-related signature

**Figure 5:**
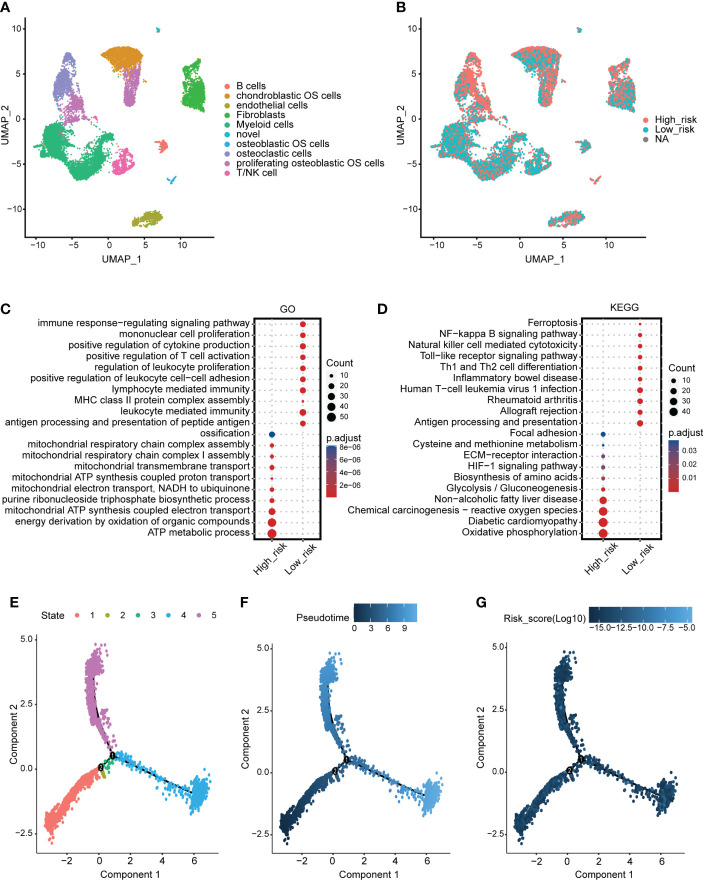
scRNA-seq analysis for the mitochondria-related signature. **(A)** The identified cells in the tumor microenvironment of osteosarcoma. **(B)** The levels of the mitochondria-related signature score in identified cells. **(C)** GO enrichment analysis for the DEGs between the two mitochondria-related signature score groups of osteosarcoma cells. **(D)** KEGG enrichment analysis for the DEGs between the two mitochondria-related signature score groups of osteosarcoma cells. **(E)** Different cell states of the pseudotime trajectory analysis on the osteosarcoma cells. **(F)** Pseudotime pattern of the pseudotime trajectory analysis on the osteosarcoma cells. **(G)** The mitochondria-related signature score of the pseudotime trajectory analysis on the osteosarcoma cells.

The identified cells in the tumor microenvironment of osteosarcoma are shown in **Figure 5A**. The levels of the mitochondria-related signature score in identified cells are shown in **Figure 5B**. The DEGs between the two mitochondria-related signature score groups of osteosarcoma cells were identified. GO enrichment analysis was performed on the DEGs (**Figure 5C**). ATP metabolic process, ossification, and energy derivation by oxidation of organic compounds were highly enriched in the high score group. Immune response-regulating signaling pathway, mononuclear cell proliferation, and positive regulation of T cell activation were highly enriched in the low score group. KEGG enrichment analysis was performed on the DEGs (**Figure 5D**). Oxidative phosphorylation, chemical carcinogenesis-reactive oxygen species, and glycolysis/gluconeogenesis were highly enriched in the high score group. Ferroptosis, Th1 and Th2 cell differentiation, and natural killer cell mediated cytotoxicity were highly enriched in the low score group. The pseudotime trajectory analysis was performed on the osteosarcoma cells, and five cell states were determined (**Figure 5E**). As pseudotime increased (**Figure 5F**), osteosarcoma cells tended to have increased mitochondria-related signature scores (**Figure 5G**). The DEGs between osteosarcoma cells around branch point 1 were identified and clustered into four types (**Figure S3A**). GO enrichment analysis was performed on the DEGs in four clusters (**Figure S3B-S3E**). The expression pattern of the mitochondria-related genes in different cell states is shown in **Figure S4**.

### Cell communication pattern of the mitochondria-related signature

**Figure 6:**
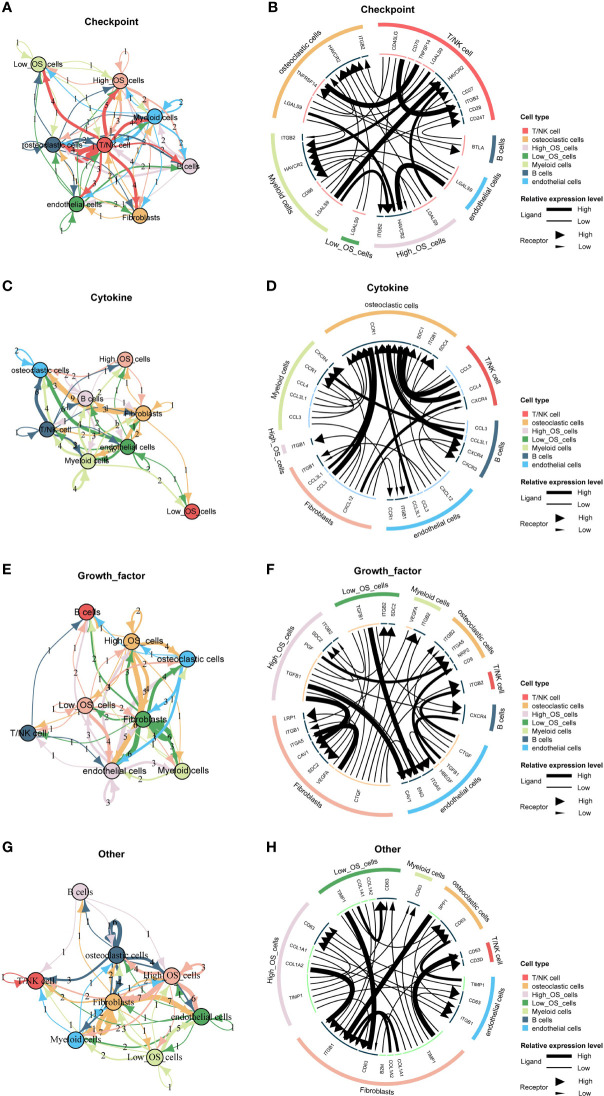
Cell communication pattern of the mitochondria-related signature. **(A)** Different cellular signaling pathways regarding checkpoints between two mitochondria-related signature score groups of osteosarcoma cells and microenvironment cells. **(B)** Cell communication pattern regarding checkpoints between two mitochondria-related signature score groups of osteosarcoma cells and microenvironment cells. **(C)** Different cellular signaling pathways regarding cytokine between two mitochondria-related signature score groups of osteosarcoma cells and microenvironment cells. **(D)** Cell communication pattern regarding cytokine between two mitochondria-related signature score groups of osteosarcoma cells and microenvironment cells. **(E)** Different cellular signaling pathways regarding growth factor between two mitochondria-related signature score groups of osteosarcoma cells and microenvironment cells. **(F)** Cell communication pattern regarding growth factor between two mitochondria-related signature score groups of osteosarcoma cells and microenvironment cells. **(G)** Different cellular signaling pathways regarding other between two mitochondria-related signature score groups of osteosarcoma cells and microenvironment cells. **(H)** Cell communication pattern regarding other between two mitochondria-related signature score groups of osteosarcoma cells and microenvironment cells.

Different cellular signaling pathways regarding checkpoints between two mitochondria-related signature score groups of osteosarcoma cells and microenvironment cells are shown in **Figures 6A, B**, in which ITGB2, HAVCR2, and LGALS9 were the most active signaling pathways in osteosarcoma cells with the high mitochondria-related signature score. Different cellular signaling pathways regarding cytokine between two mitochondria-related signature score groups of osteosarcoma cells and microenvironment cells are shown in **Figures 6C, D**, in which ITGB1 was the most active signaling pathway in osteosarcoma cells with the high mitochondria-related signature score. Different cellular signaling pathways regarding growth factor between two mitochondria-related signature score groups of osteosarcoma cells and microenvironment cells are shown in **Figures 6E, F**, in which ITGB2, SDC2, PGF, and TGFB1 were the most active signaling pathways in osteosarcoma cells with the high mitochondria-related signature score. Different cellular signaling pathways regarding other between two mitochondria-related signature score groups of osteosarcoma cells and microenvironment cells are shown in **Figures 6G, H**, in which CD63, COL1A1, COL1A2, and TIMP1 were the most active signaling pathways in osteosarcoma cells with the high mitochondria-related signature score.

### *In vitro* validation on PCCB

**Figure 7:**
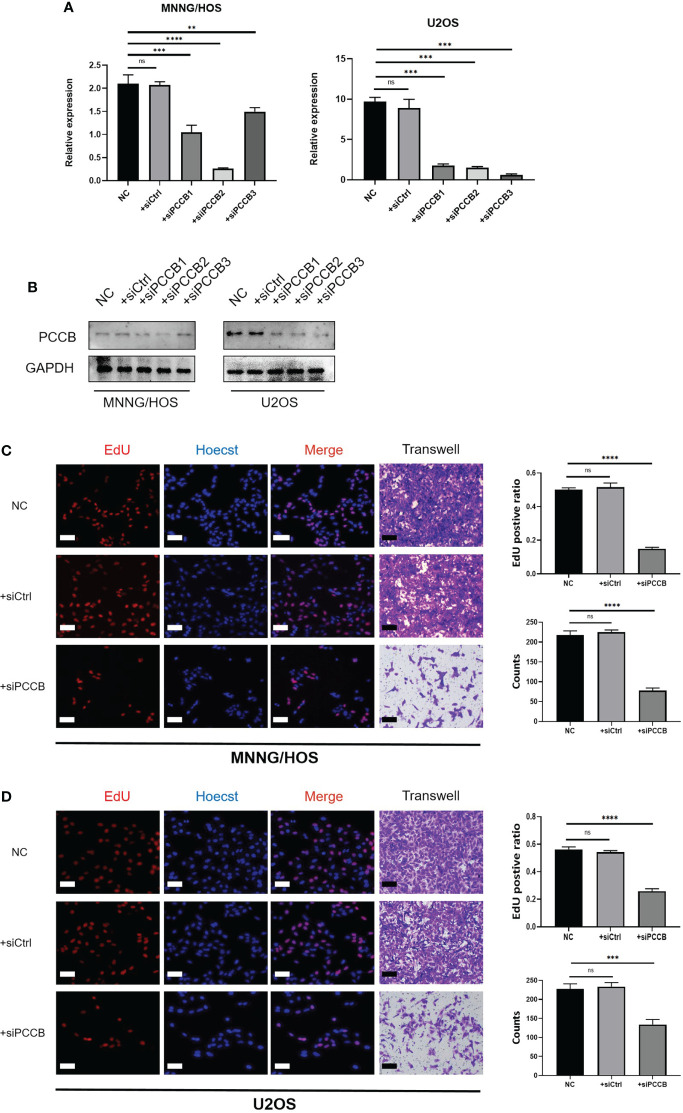
*In vitro* validation on PCCB. **(A)** RT-qPCR results of the expression of PCCB in five groups (NC, si-NC, si-PCCB-1, si-PCCB-2, si-PCCB-3) in two cell lines. **(B)** Western blot results of the expression of PCCB in five groups (NC, si-NC, si-PCCB-1, si-PCCB-2, si-PCCB-3) in two cell lines. **(C)** EdU assay in three groups (NC, si-NC, si-PCCB) in the MNNG/HOS cell line. Statistical analysis was based on mean ± SD. **(D)** EdU assay in three groups (NC, si-NC, si-PCCB) in the U2OS cell line. Statistical analysis was based on mean ± SD. The si-PCCB refers to siRNA transfection of PCCB. The si-NC refers to siRNA transfection of nonspecific control. ns, no significance; **p < 0.01; ***p < 0.001;****p < 0.0001.

**Figure 8:**
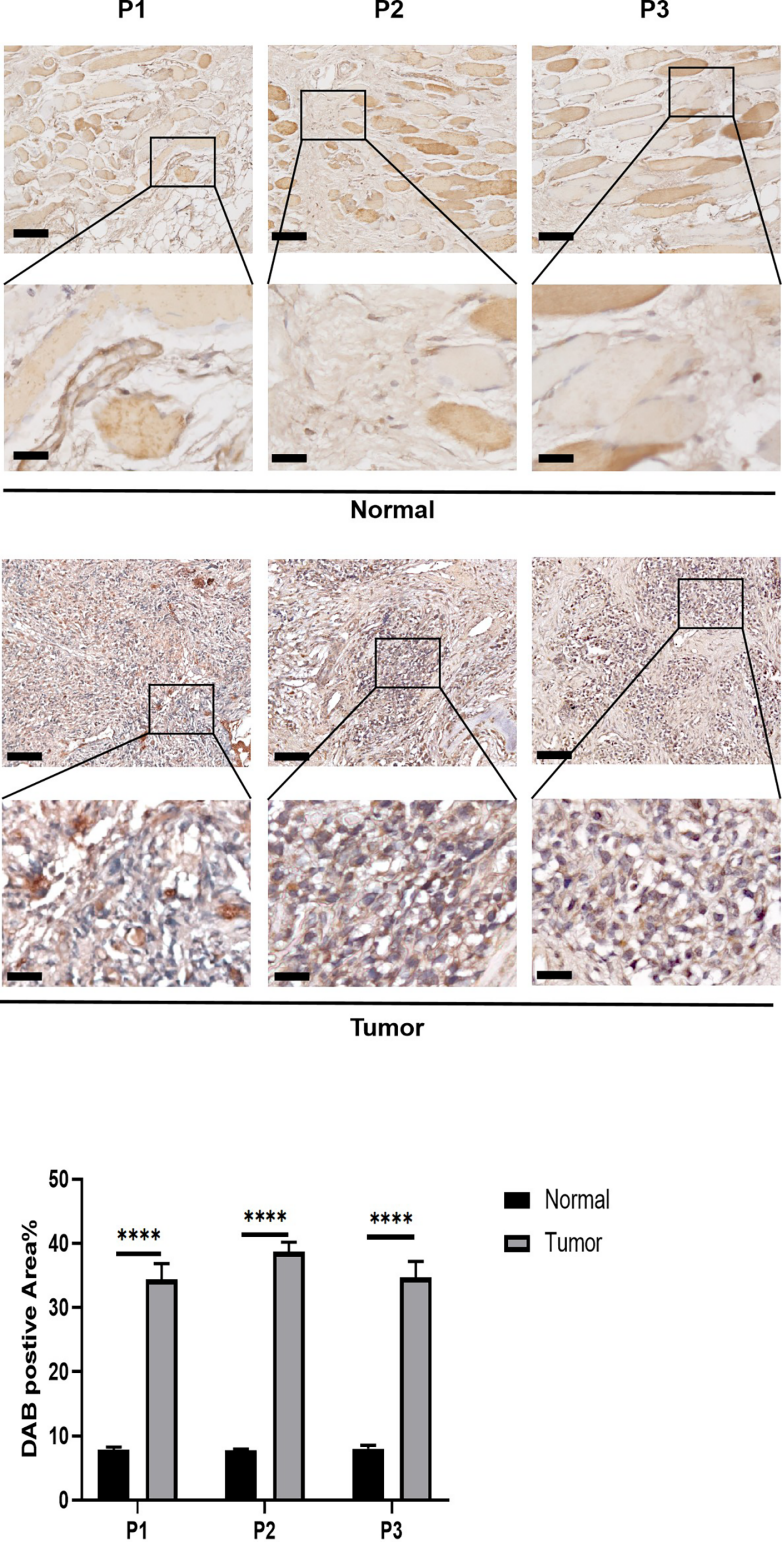
*In vitro* validation on PCCB. IHC results of the expression of PCCB in osteosarcoma tumor tissues and normal tissues. The upper row is 10x images of the sections, and the lower row is 40x images of the sections. Statistical analysis was based on mean ± SD. ****p < 0.0001.

The expression pattern of PCCB in the identified cells in the tumor microenvironment of osteosarcoma is shown in **Figures S5A-S5C**, in which PCCB was highly expressed by osteosarcoma cells. GO enrichment analysis revealed that DNA replication, sterol biosynthetic process, and cholesterol biosynthetic process were highly enriched in osteosarcoma patients with high expression of PCCB. In contrast, T cell activation, B cell activation, and lymphocyte proliferation were highly enriched in osteosarcoma patients with low expression of PCCB (**Figure S5D**). KEGG enrichment analysis revealed that cell cycle, carbon metabolism, and biosynthesis of amino acids were highly enriched in osteosarcoma patients with high expression of PCCB. In contrast, Th1 and Th2 cell differentiation, T cell receptor signaling pathway, and B cell receptor signaling pathway were highly enriched in osteosarcoma patients with low expression of PCCB (**Figure S5E**). As the most hazardous gene based on the stepwise multivariate Cox regression analysis, the biological function of PCCB in osteosarcoma was explored. RT-qPCR (**Figure 7A**) and western blot (**Figure 7B**) results showed that the expression of PCCB was significantly inhibited in three si-PCCB groups compared to NC and si-NC groups in U2OS and MNNG/HOS cells. The si-PCCB, with the most vital ability to suppress the expression of PCCB, was used for the follow-up experiment. EdU assay revealed that the proliferation ability of U2OS (**Figure 7D**) and MNNG/HOS (**Figure 7C**) cells was significantly inhibited after transfection with si-PCCB. The IHC results further confirmed that the expression of PCCB was considerably higher in osteosarcoma tumor tissues than in normal tissues (**Figure 8**).

### Immunotherapy prediction of PCCB

**Figure 9:**
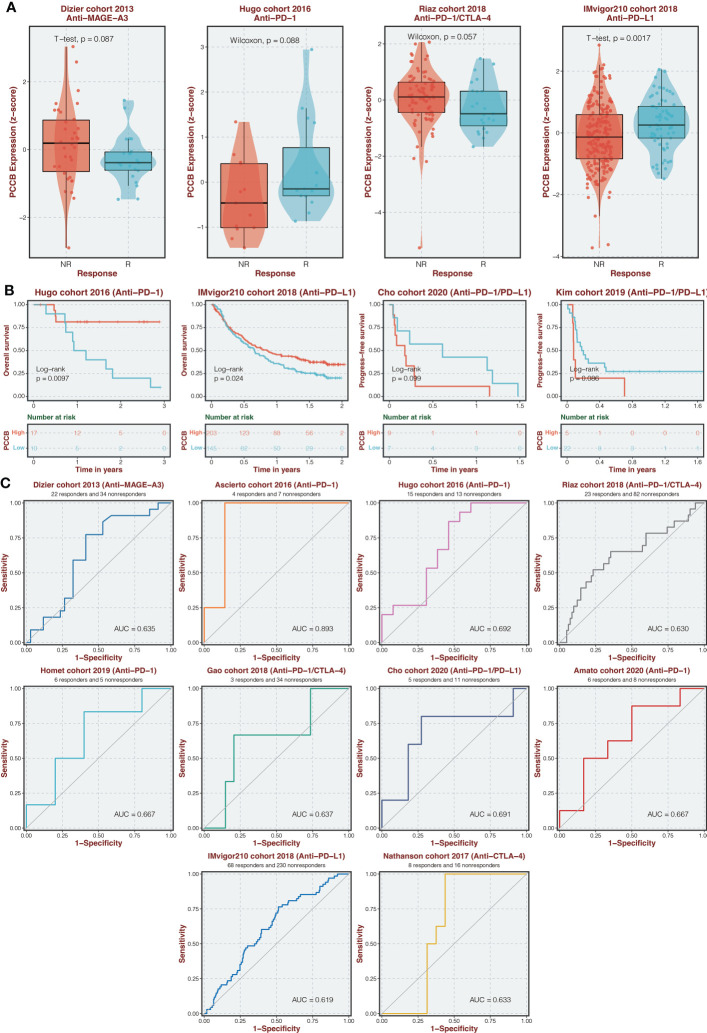
Immunotherapy prediction of PCCB. **(A)** The expression of PCCB in responders and non-responders in immunotherapy cohorts. **(B)** Survival analysis was performed on the two groups regarding PCCB expression in immunotherapy cohorts. **(C)** The ROC curve of PCCB in predicting immunotherapy response in immunotherapy cohorts.

The expression of PCCB in responders and non-responders in immunotherapy cohorts is shown in **Figure 9A**, in which non-responders had higher expression of PCCB in the Dizier cohort 2013 and Riaz cohort 2018 while responders had higher expression of PCCB in the Hugo cohort 2016 and IMvigor210 cohort 2018. Survival analysis was performed on the two groups regarding PCCB expression in immunotherapy cohorts (**Figure 9B**). PCCB was associated with better survival in the Hugo cohort 2016 and IMvigor210 cohort 2018, while PCCB was associated with worse survival in the Cho cohort 2020 and Kim cohort 2019. PCCB showed potent efficacy in predicting immunotherapy response in ten immunotherapy cohorts (**Figure 9C**).

## Discussion

In the big data era, mining potential diagnostic, prognostic, and predictive markers in cancer based on large-scale bioinformatics analysis has been increasingly important. Many established markers showed great potential in clinical application (17, 18). Mitochondrial dysfunction is known as a hallmark of cancer. Briefly, mitochondrial dysfunction can be caused by mtDNA mutation, oxidative stress, defective mitochondrial electron transport chain, defective mitochondrial TCA cycle enzyme, tumor-promoting signals, etc (19). The result is the change of cell metabolic pathway, the destruction of intracellular REDOX homeostasis, and the generation of apoptosis and drug resistance. Finally, mitochondrial dysfunction would lead to genomic instability, the aging process, and the occurrence and development of cancer (19). Mitochondria dysfunction in CD8+ T cells has also been demonstrated to be an essential contributing factor for cancer development and a potential target for cancer treatment (20). Therefore, an in-depth understanding of mitochondrial dysfunction in cancer is essential for developing novel effective therapeutic strategies. Most studies have focused on the pathogenic molecular mechanisms of individual mitochondria-related genes. Although several studies have comprehensively explored the potential values of mitochondria-related genes in hepatocellular carcinoma, clear cell renal cell carcinoma, etc., a comprehensive evaluation of the mitochondria-related genes in osteosarcoma has never been conducted.

The current study developed a mitochondria-related signature in osteosarcoma patients based on 16 mitochondria-related genes. ACADVL was reported to be associated with the loss of heterozygosity of 17p13 in the pathogenesis of adrenocortical tumors (21). OGDH was a critical tumor promoter in cancer (22). TRMT1 was found to serve as a promising biomarker in clear cell renal cell carcinoma (23). Downregulation of mitochondrial UQCRB was reported to inhibit cancer stem cell-like properties in glioblastoma (24). FDX1 was revealed to impact the prognosis and mediate the metabolism of lung adenocarcinoma (25). EPHX2 could inhibit colon cancer progression by promoting fatty acid degradation (26). STOML2 was reported to potentiate metastasis of hepatocellular carcinoma by promoting PINK1-mediated mitophagy and regulating sensitivity to Lenvatinib (27). Glycolysis gatekeeper PDK1 could reprogram breast cancer stem cells under hypoxia (28). ALDH7A1 knockdown significantly reduces tumor formation in pancreatic ductal adenocarcinoma (29). LACTB could suppress melanoma progression by attenuating PP1A and YAP interaction (30). miR-125b/BAK1 pathway was essential in promoting tumorigenesis and inhibiting apoptosis of cervical cancer (31). MFN2 could suppress cancer progression by inhibiting mTORC2/Akt signaling (32).

As the most hazardous gene in the mitochondria-related signature, PCCB was found to mediate the proliferation of proliferation and migration of osteosarcoma cells. Besides, PCCB was found with significantly higher expression in osteosarcoma tumor tissues than in normal tissues. Therefore, PCCB was a potential oncogene in osteosarcoma.

Despite different clinical factors (age, gender, metastasis), Osteosarcoma patients with high mitochondria-related signature scores presented decreased survival time. Besides, the mitochondria-related signature was associated with tumor metastasis. Therefore, the mitochondria-related signature was a potential prognostic marker in osteosarcoma patients. Besides, osteosarcoma patients with high mitochondria-related signature scores were found with a relatively immune cold microenvironment, indicating the un-suppressed malignancy of the tumor. The functional annotation of the mitochondria-related signature further confirmed that the tumorigenic pathways were more active in osteosarcoma patients with high mitochondria-related signature scores.

In contrast, the immunogenic pathways were more involved in osteosarcoma patients with low mitochondria-related signature scores. The potential value of the mitochondria-related signature in predicting chemotherapy agents was also confirmed. 24 drugs were negatively associated with the mitochondria-related signature, including apoptosis regulation inhibitor AZD5991, protein stability and degradation inhibitor ML323, and kinases inhibitor BMS-345541. In addition, six drugs were positively associated with the mitochondria-related signature, including ERK MAPK signaling inhibitor Refametinib, RTK signaling inhibitor NVP-TAE684, and kinases inhibitor A-770041.

At the scRNA-seq level, osteosarcoma cells gradually evolved into tumors with high mitochondria-related signature scores. The functional annotation of the mitochondria-related signature also confirmed the active tumorigenic pathways and inactive immunogenic pathways in osteosarcoma cells with high mitochondria-related signature scores. The tumor microenvironment has already been proven to essentially influence the proliferation, migration, and invasion of cancer (33, 34). Checkpoints (ITGB2, HAVCR2, and LGALS9), cytokine (ITGB1), growth factor (ITGB2, SDC2, PGF, and TGFB1), and other (CD63, COL1A1, COL1A2, and TIMP1) were the most active signaling pathways involved in the cell communication between osteosarcoma cells with a high mitochondria-related signature score and microenvironment cells, indicating the potential immune evasion and tumor progression in osteosarcoma cells with a high mitochondria-related signature score.

## Conclusion

Taken together, a mitochondria-related signature was developed in osteosarcoma with robust predictive values in the immune microenvironment, chemotherapy agents, and prognosis. The potential clinical application of the mitochondria-related signature is expected to be further validated by real-world cohorts. PCCB was a potential oncogene in osteosarcoma, and the related complex regulatory mechanisms remain to be further explored.

## Data availability statement

Publicly available datasets were analyzed in this study. This data can be found here: TARGET-OS(https://xenabrowser.net/) and GEO (https://www.ncbi.nlm.nih.gov/geo).

## Ethics statement

The studies involving human participants were reviewed and approved by the institutional review board (IRB) of the Third Xiangya Hospital, Central South University. Written informed consent for participation was not required for this study in accordance with the national legislation and the institutional requirements.

## Author contributions

LZ conceived and performed most of the experiments. JH wrote the manuscript. YS and YY collected and analyzed the data. XC provided experimental advice and supervised the study; SW provided funding support. All authors read and approved the final manuscript.

## Funding

This work was supported by the National Natural Science Foundation of China (82072501), Science and Technology Innovation Leading Plan of High Tech Industry in Hunan Province (2020SK2011).

## Conflict of interest

The authors declare that the research was conducted in the absence of any commercial or financial relationships that could be construed as a potential conflict of interest.

## Publisher’s note

All claims expressed in this article are solely those of the authors and do not necessarily represent those of their affiliated organizations, or those of the publisher, the editors and the reviewers. Any product that may be evaluated in this article, or claim that may be made by its manufacturer, is not guaranteed or endorsed by the publisher.

## Abbreviations

TARGET, Therapeutically Applicable Research to Generate Effective Treatments; GEO, Gene Expression Omnibus; scRNA-seq, single-cell RNA sequencing; mtROS, mitochondrial reactive oxygen species; LASSO, least absolute shrinkage and selection operator; ROC, receiver operating characteristic; AUC, area under the curve; ssGSEA, single-sample gene-set enrichment analysis; ESTIMATE, Estimated Stromal and Immune cells in Malignant Tumor tissues using Expression data; DEGs, differentially expressed genes; GSVA, gene set variation analysis; GO, Gene Ontology; KEGG, Kyoto Encyclopedia of Genes and Genomes; IHC, Immunohistochemistry; RT-qPCR, Real-time quantitative polymerase chain reaction.
