# Supplementary material for: A mitochondria-related signature for predicting immune microenvironment and therapeutic response in osteosarcoma
Source: Front Oncol. 2022 Dec 1;12:1085065. doi: 10.3389/fonc.2022.1085065 (PMC9751795; doi:10.3389/fonc.2022.1085065)
Supplement: Supplementary file 1 [file DataSheet_1.docx]

Supplementary Figures


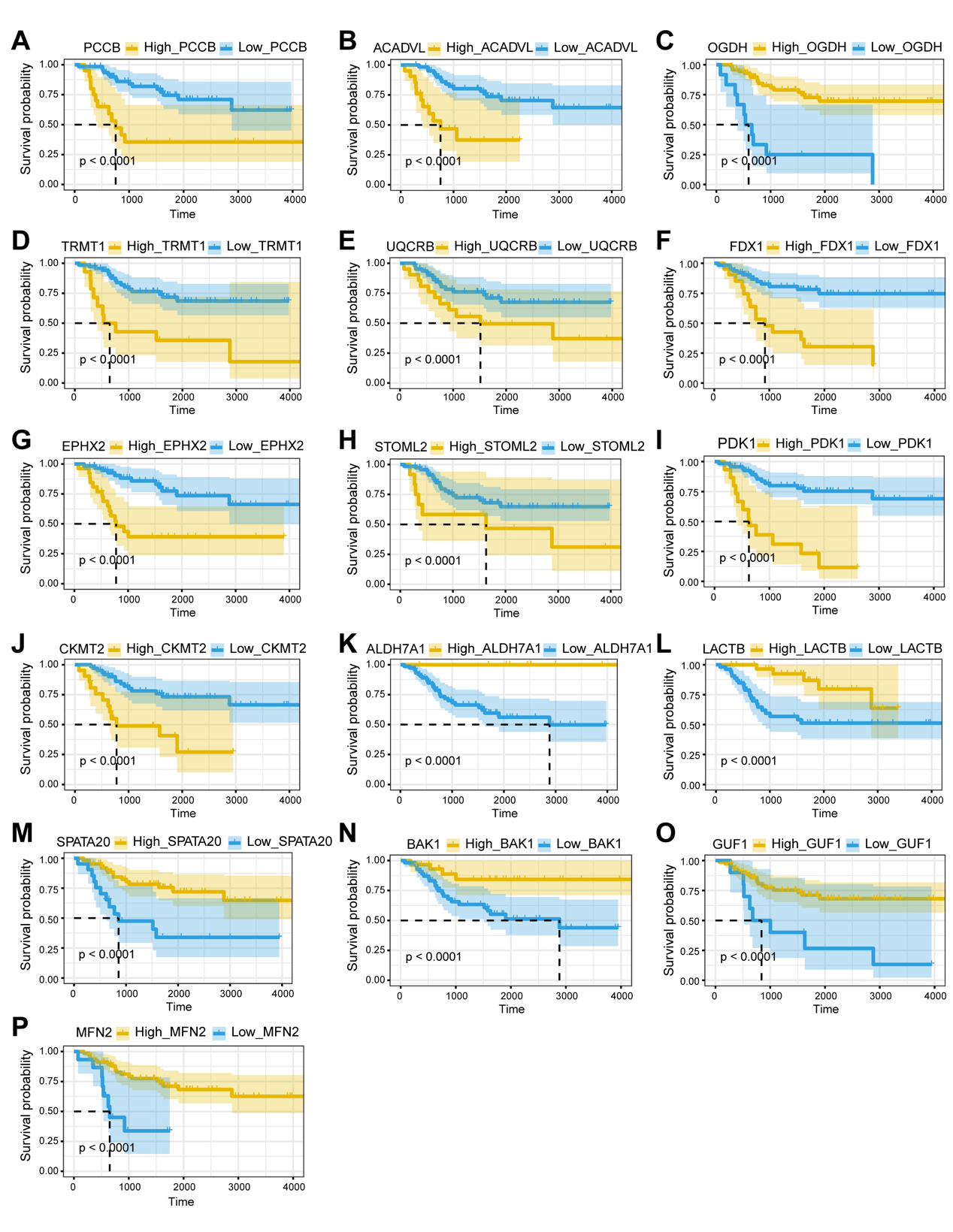


Figure S1. Prognostic value of the mitochondria-related genes.


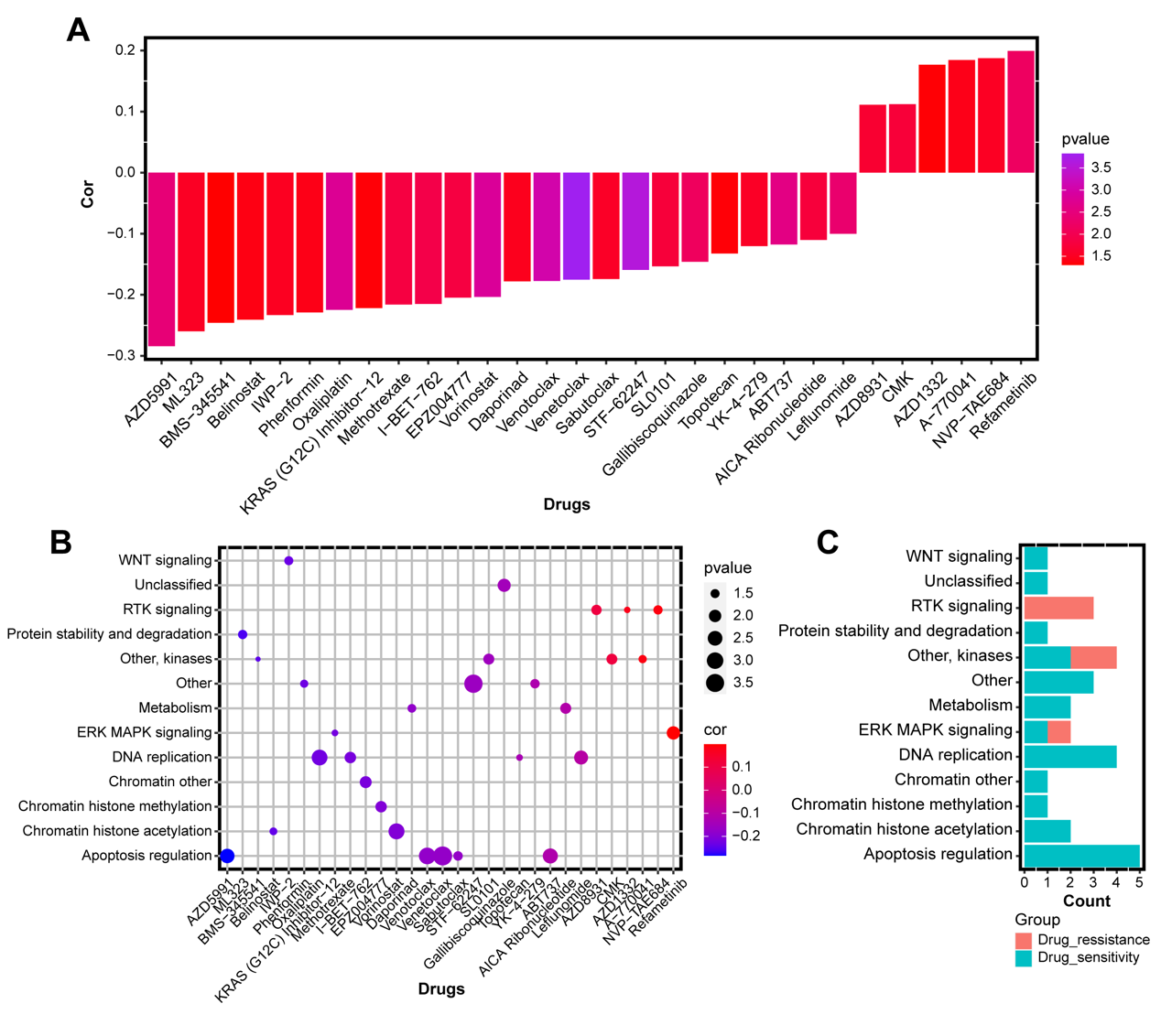


Figure S2. Predictive value of the mitochondria-related signature in drug sensitivity and resistance in GSDC. A. Bar diagram of a correlation between IC50 of anti-cancer drugs and the mitochondria-related signature scores. Altitude represents the correlation, the higher the altitude, the higher the correlation. Color represents statistical significance (p value), the more purple the color, the greater the significance. B. Scatter diagram of a correlation between targeted signaling pathways and IC50 of significant anti-cancer drugs. The size of plots represents statistical significance (p value), the larger the size, the greater the significance. The color of the plots represents the correlation between targeted pathways and anti-cancer drugs. Red represents a positive correlation and blue represents a negative correlation. Purple represents little correlation. C. Bar diagram shows the counts of sensitive drugs and resistant drugs regarding the targeted pathways.


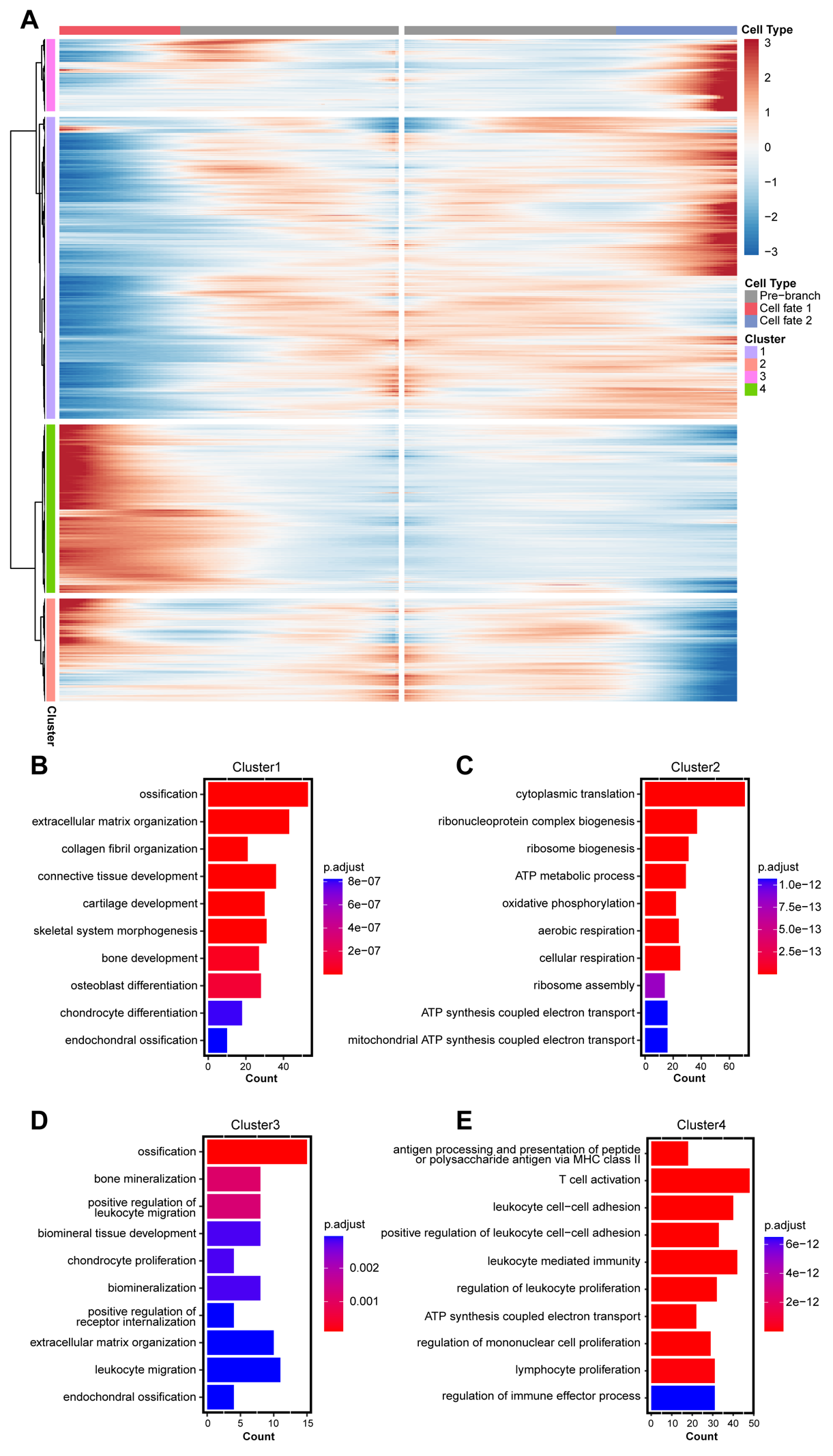


Figure S3. Functional annotation of the mitochondria-related signature at the scRNA-seq level. A. Heatmap of the DEGs between osteosarcoma cells around branch point 1. B. GO enrichment analysis for the DEGs in cluster 1. C. GO enrichment analysis for the DEGs in cluster 2. D. GO enrichment analysis for the DEGs in cluster 3. E. GO enrichment analysis for the DEGs in cluster 4.


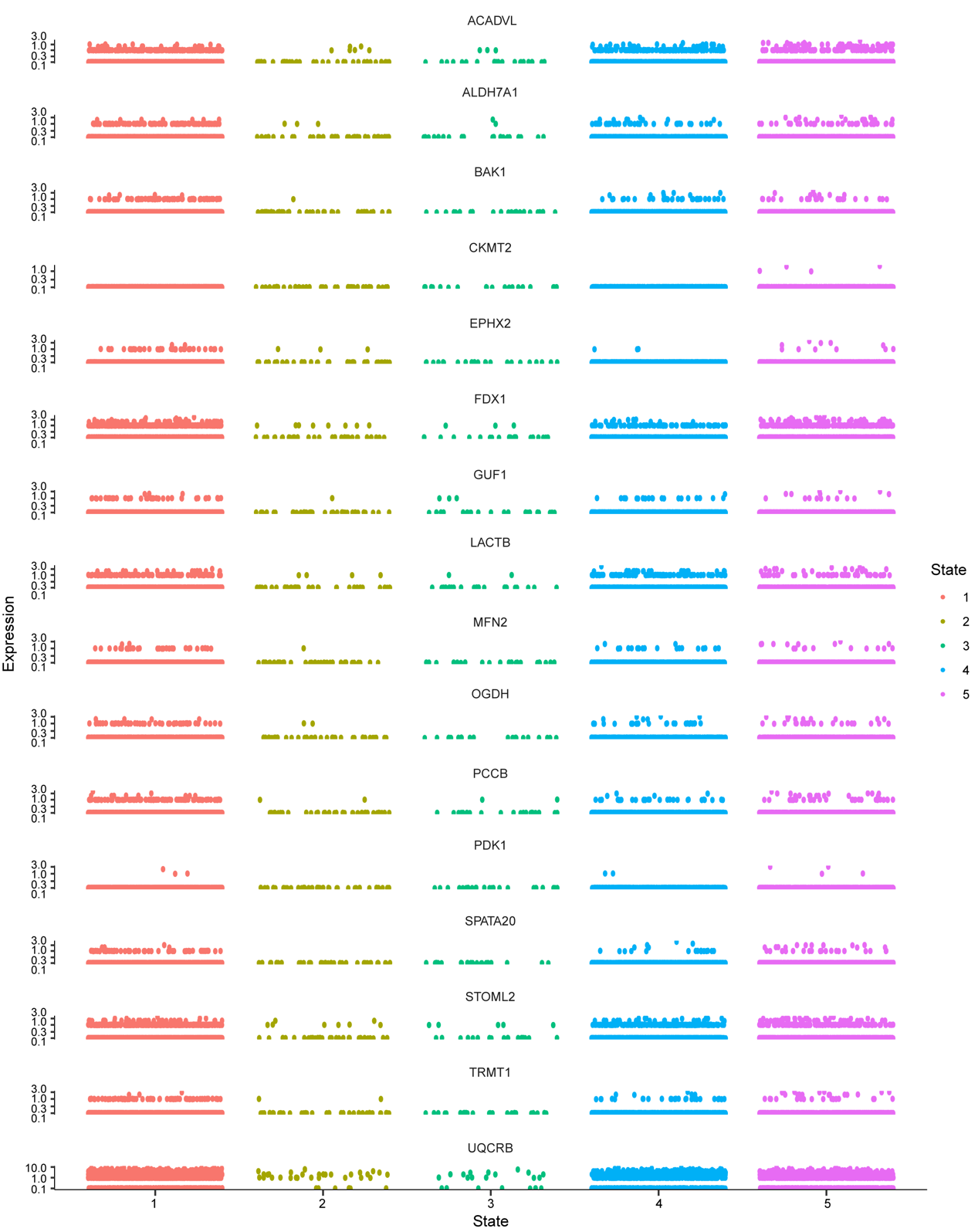


Figure S4. The expression pattern of the mitochondria-related genes in different cell states.


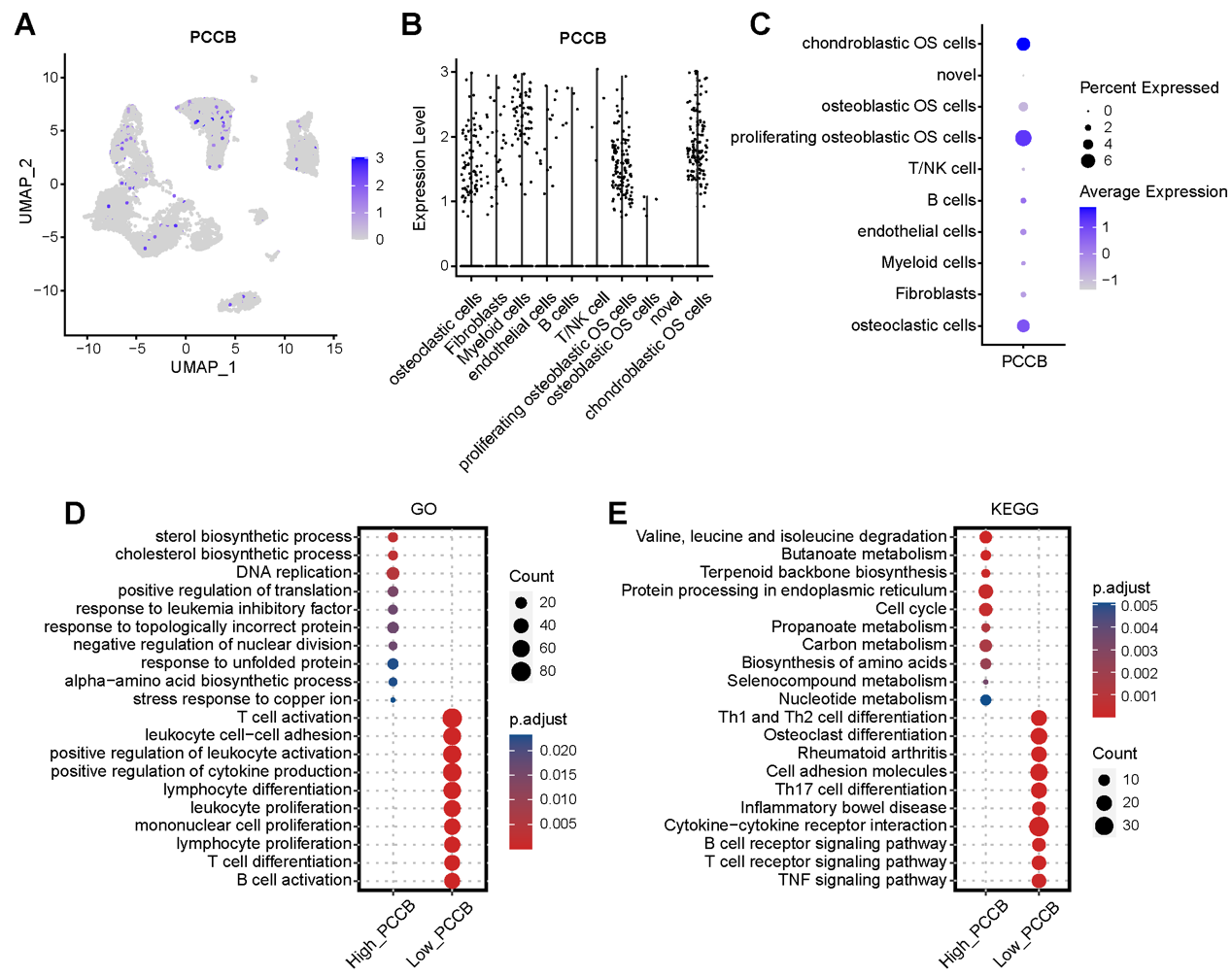


Figure S5. The molecular feature of PCCB. A. The expression of PCCB in the tumor microenvironment of osteosarcoma based on the UMAP dimension. B. The expression of PCCB in identified cells in the tumor microenvironment of osteosarcoma based on Vlnplot. C. The expression of PCCB in identified cells in the tumor microenvironment of osteosarcoma based on Dotplot. D. GO enrichment analysis on PCCB. E. KEGG enrichment analysis on PCCB.
